# A Patient with Generalized Weakness – A Case Report

**DOI:** 10.21980/J8593C

**Published:** 2023-07-31

**Authors:** Darby Graham, Manparbodh Kaur, John Costumbrado, Sassan Ghassemzadeh

**Affiliations:** * University of California, Riverside, School of Medicine, Riverside, CA; ^ Riverside Community Hospital, Department of Emergency Medicine, Riverside, CA

## Abstract

Emphysematous pyelonephritis (EPN) is a rare and life-threatening sequalae of a urinary tract infection marked by a gas-producing necrotizing infection of the renal parenchyma requiring prompt treatment. It has been found to be correlated with uncontrolled diabetes mellitus and is more common in females due to their increased susceptibility to urinary tract infections. Early recognition and treatment of EPN are essential to improve the patient’s prognosis and prevent further complications. In this case we highlight a progressive case of pyelonephritis complicated by emphysematous abscess formation requiring surgical management. A 49-year-old female with a history of diabetes, hypertension, asthma, and lupus who presented to the emergency department (ED) for evaluation of generalized weakness. Lactate was elevated and urinalysis was suggestive of infection. A computed tomography (CT) scan of the abdomen and pelvis revealed a large subcapsular fluid collection with multiple gas and air-fluid levels along the right kidney, as well as another suspected fluid collection adjacent to the upper pole of the right kidney. The findings suggested complicated pyelonephritis with emphysematous abscess formation. Urology was consulted and they suggested a perinephric drain be placed by interventional radiology (IR). The patient progressed well and was ultimately discharged days later in stable condition. Despite its rarity, healthcare providers should consider infectious processes such as EPN in the differential for generalized weakness and promptly initiate appropriate diagnostic and therapeutic measures.

**Topics:**

Weakness, sepsis, urology, CT scan.

[Fig f1-jetem-8-3-v14] [Fig f2-jetem-8-3-v14]

**Figure f1-jetem-8-3-v14:**
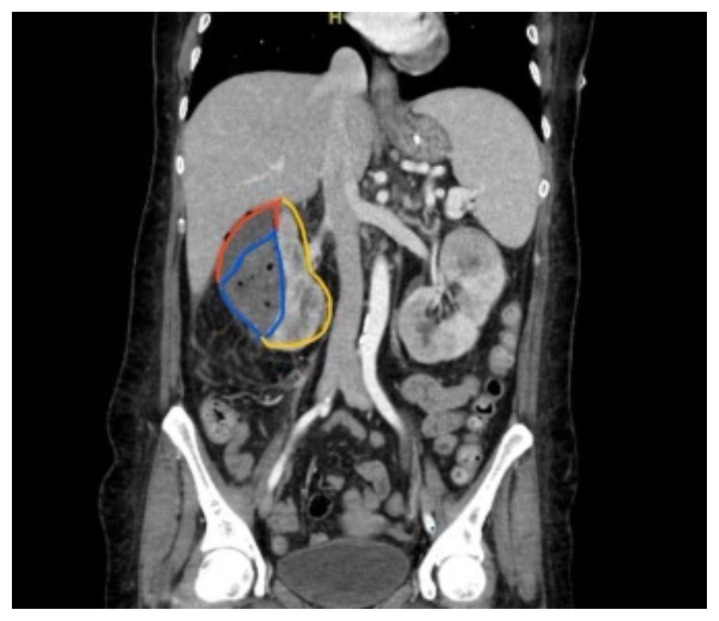


**Figure f2-jetem-8-3-v14:**
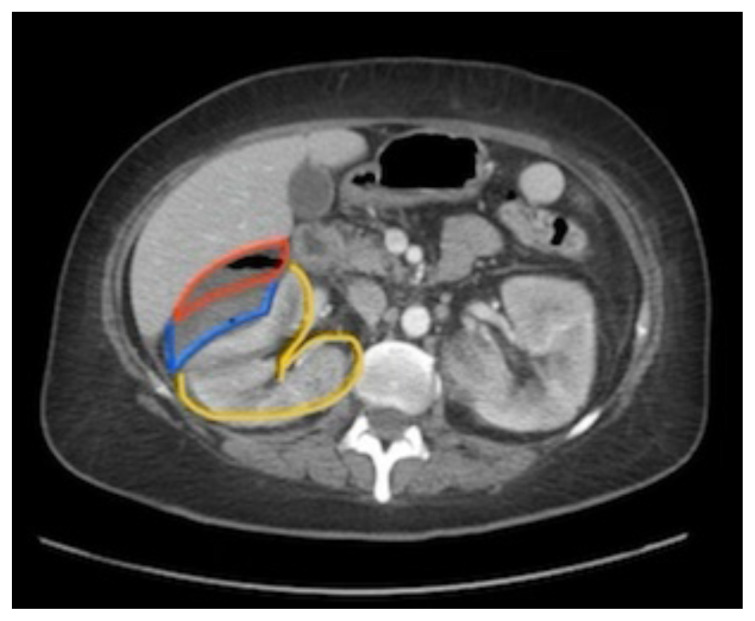


## Brief introduction

Generalized weakness is a common ED complaint that is challenging due to varied presentations. Additionally, it has a broad differential including infectious, neurological, endocrinological, cardiovascular, and other etiologies. We report a case of generalized weakness due to EPN complicated by subcapsular hematoma formation and its pharmacological and surgical management. Emphysematous pyelonephritis is a rare and life-threatening complication of urinary tract infections (UTIs). It is characterized by a gas-producing necrotizing infection of the renal parenchyma, which can rapidly lead to septicemia and multi-organ failure. EPN is typically caused by a bacterial infection, most commonly by *Escherichia coli* or *Klebsiella pneumoniae*, and is most frequently observed in patients with diabetes mellitus or other immunocompromising conditions.^1^ The symptoms of EPN can vary from mild to severe, depending on the extent and severity of the infection. Patients may experience fever, chills, nausea, vomiting, abdominal pain, and difficulty passing urine. In severe cases, the infection can cause septic shock, respiratory failure, and even death.

Emphysematous pyelonephritis is a medical emergency that requires prompt diagnosis and treatment. Imaging tests such as CT scan and ultrasound can help to confirm the diagnosis. The presence of gas in the renal parenchyma on radiological imaging is highly suggestive of EPN, although other conditions such as emphysematous cystitis and emphysematous pyelitis should be considered in the differential diagnosis.^2^ The management of EPN typically involves prompt initiation of broad-spectrum antibiotics, aggressive hydration, and close monitoring of vital signs and laboratory parameters. In some cases, surgical intervention may be necessary, such as percutaneous catheter drainage or nephrectomy to prevent the spread of the infection. Management with percutaneous catheter drainage has been found to decrease mortality rates in patients with EPN and is the current treatment of choice.^3^,^4^

## Presenting concerns and clinical findings

The patient is a 49-year-old female with a history of poorly-controlled diabetes, hypertension, asthma, and lupus who presented to the ED for evaluation of generalized weakness. She reports symptoms for the past month and was recently hospitalized at an outside hospital for severe hypokalemia. She also endorses non-compliance with her diabetic medications. Her symptoms worsened with new nausea and vomiting, which prompted the current ED visit. She had no focal weakness or sensory changes. She initially presented with vitals within normal limits and no complaints of pain by history or on physical exam. Other than skin pallor, her exam was unremarkable. After the patient returned from using the restroom to give a urine sample, she developed diffuse abdominal pain and nausea. She also became tachypneic and tachycardic. Sepsis protocols were initiated and 30 mL/kg bolus of crystalloids, appropriate cultures, and 2 gm of intravenous (IV) ceftriaxone were ordered. Initial lab results were significant for a white blood cell count of 4.7 K/mm3 and hemoglobin of 6.8 gm/dL. One unit of packed red blood cells ordered. Diabetic ketoacidosis was considered with the patient’s hyperglycemia at 349 mg/dL and elevated beta-hydroxybutyrate level; however, serum bicarbonate level and pH on a venous blood gas were within normal limits. Lactate was elevated to 2.9 mmol/L and urinalysis was suggestive of infection. Severe sepsis due to complicated urinary tract infection was suspected. Patient was immediately transported to imaging for a CT scan of the abdomen and pelvis with IV contrast.

## Significant findings

The CT of the abdomen and pelvis showed evidence of a large subcapsular rim-enhancing fluid collection with multiple gas and air-fluid levels along the right kidney measuring 8 × 4 cm axially and 11 cm craniocaudally (blue outline) with mass effect on the right renal parenchyma (yellow outline). Another suspected fluid collection adjacent to the upper pole of the right kidney measuring 4 × 3.4 cm was noted (red outline). Bilateral pyelonephritis was suggested without hydronephrosis or nephrolithiasis. The findings suggested complicated pyelonephritis with emphysematous abscess and hematoma formation.

## Patient course

While the patient initially presented in stable condition, her condition deteriorated rapidly. Shortly after re-evaluation, sepsis management was initiated and CT of the abdomen and pelvis with IV contrast was obtained. Given the findings of bilateral complicated pyelonephritis with emphysematous abscess formation in the context of severe sepsis, urology was consulted and the patient was admitted to the intermediate care unit.

Urology recommended broadening antibiotic coverage and drainage of the suspected abscess and hematoma by interventional radiology (IR). Coverage was broadened with cefepime, metronidazole, and vancomycin and an aggressive sliding scale insulin regimen was added for her hyperglycemia. IR was consulted, and subsequently, a perinephric drain was placed with approximately 30 mL of sanguineous fluid removed. Given the presence of gas in the fluid collections seen on CT, the drain was left in place and the patient continued to have about 200 mL of drainage a day while in the hospital. The drainage sample sent for culture resulted positive for *Klebsiella pneumoniae*, which also grew in the blood and urine cultures. Given the sensitivities of the cultures, the antibiotic coverage was narrowed to ceftriaxone in conjunction with infectious disease (ID) recommendations.

The patient’s symptoms and vitals improved over the course of the hospitalization, and she was discharged after nine days in stable condition on oral ciprofloxacin with the drain left in place, due to continued output, and urology outpatient follow-up for removal.

## Discussion

Emphysematous pyelonephritis is a rare necrotizing infection that is primarily observed in diabetic women, with a female-to-male ratio of 3:1.^5^ The increased incidence of EPN in females may be attributed to their higher susceptibility to urinary tract infections.^6^ Uncontrolled diabetes mellitus and urinary tract obstruction are the major risk factors associated with EPN. The occurrence of EPN is almost exclusively (90%) observed in individuals with diabetes, with *Escherichia coli* (70%), *Klebsiella pneumoniae* (29%), and *Proteus* being the most common pathogens.^3^ The pathogenesis of EPN is believed to be attributed to various factors, including gas-forming bacteria, elevated tissue glucose concentrations, impaired tissue perfusion, and weakened immune response, which is commonly observed in individuals with diabetes mellitus.^7^

While our patient in this case report did not exhibit the classic signs and symptoms of EPN, including fever/chills, flank pain, renal angle tenderness, vomiting, and dysuria, these are the most reported clinical features.^8^ However, these symptoms and signs are nonspecific and cannot be used to differentiate EPN from typical pyelonephritis. The presence of crepitus in the lumbar region is an extremely rare finding but can serve as a crucial clinical indicator of EPN.^8^ In contrast to our reported case, the left kidney has been found to be more frequently involved than the right.^3^

In terms of diagnostic imaging, renal ultrasound has a diagnostic accuracy of approximately 80% for confirming the presence of EPN.^9^ Although abdominal plain films can be used as the primary imaging modality, gas may only be visualized in around 33% of cases and may be indistinguishable from air in the bowel.^3^ If there is a high suspicion of EPN, a CT scan is necessary because it is 100% sensitive.^10^ Therefore, a CT scan is recommended for the accurate diagnosis of EPN and may also guide its treatment.

The Huang-Tseng CT-based classification system identifies the following categories based on the extension of gas: (1) class 1: gas in the collecting system only (so-called emphysematous pyelitis), (2) class 2: gas in the renal parenchyma without extension to the extrarenal space, (3) class 3A: extension of gas or abscess to the perinephric space, class 3B: extension of gas or abscess to the pararenal space, and (4) class 4: bilateral EPN or solitary kidney with EPN.^3^ The outcome of each classification of EPN is variable based on the management and intervention of the patient. Based on this classification system, the patient from our case falls into class 2. In this category, it has been found that there is the greatest survival rate with percutaneous drainage or ureteral stents in conjunction with antibiotics; however, there have been several cases reported that resulted in successful recovery without drainage.^11^

The treatment of EPN is typically divided into medical and surgical management and has become more conservative over the years. The primary goal of medical management is to control the infection and improve the patient’s overall condition. This includes the administration of broad-spectrum antibiotics to cover the usual suspects of EPN (*Escherichia coli, Klebsiella pneumoniae*, and *Proteus*), along with supportive care such as intravenous fluids, electrolyte replacement, and glucose correction.^12^ It has been recommended to treat with third or fourth generation cephalosporins, aminoglycoside, or carbapenems; however, patients risk factors and possibility of multi-drug resistance (MDR) can change the course of treatment.^13^,^14^ In a retrospective study of 72 patients with EPN, it was found that 45% of the patients had third generation cephalosporin antibiotic resistance, which was attributed to overuse of antibiotics.^14^ Ultimately the choice of antibiotics should be guided by the patient’s clinical status, culture and sensitivity results, and the endemic bacterial resistance within the current geographic location.

Surgical management is indicated in cases of severe disease or when medical management alone fails. Additionally, it has been found that shock, thrombocytopenia, confusion, and hyponatremia are risk factors that lead to increased mortality of patients with EPN and can mandate more aggressive treatments.^15^ There is evidence that the mortality was improved when using medical management combined with percutaneous drainage (13.5%) when compared with emergency nephrectomy (25%).^4^ Percutaneous drainage is the treatment of choice when kidney functioning is intact.^16^ In some cases, nephrectomy may be necessary to control the infection and prevent sepsis. This option has been reserved for patients that are classified into class III and IV as described by the Huang-Tseng CT-based classification system.^3^

Diagnosing generalized weakness as a chief complaint in the ED can be challenging due to the numerous potential differential diagnoses that involve multiple systems of the body, making it an elusive symptom to diagnose. However, infection leading to sepsis is always an important diagnosis to consider in such patients since prompt recognition and treatment can improve outcomes. While EPN is a rare diagnosis, it is a serious infection that requires CT abdomen and pelvis to guide treatment and establish a definitive diagnosis. This is due to the difficulty of differentiating pyelonephritis and EPN using other imaging modalities and physical exam. This case report underscores the importance of recognizing sepsis given the high mortality associated with severe sepsis and septic shock. In suspected sepsis, timely interventions such as fluid resuscitation, antibiotic therapy, and source control are critical considerations that came into play in this patient with EPN.

## Supplementary Information








