# Supplementary figures and images for: A Patient with Generalized Weakness – A Case Report
Source: J Educ Teach Emerg Med. 2023 Jul 31;8(3):V14–8. doi: 10.21980/J8593C (PMC10414978; doi:10.21980/J8593C)

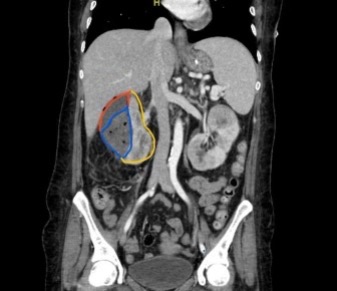

Supplement: Supplementary file 1 [file JETem-8-3-V14-supp1.jpg]

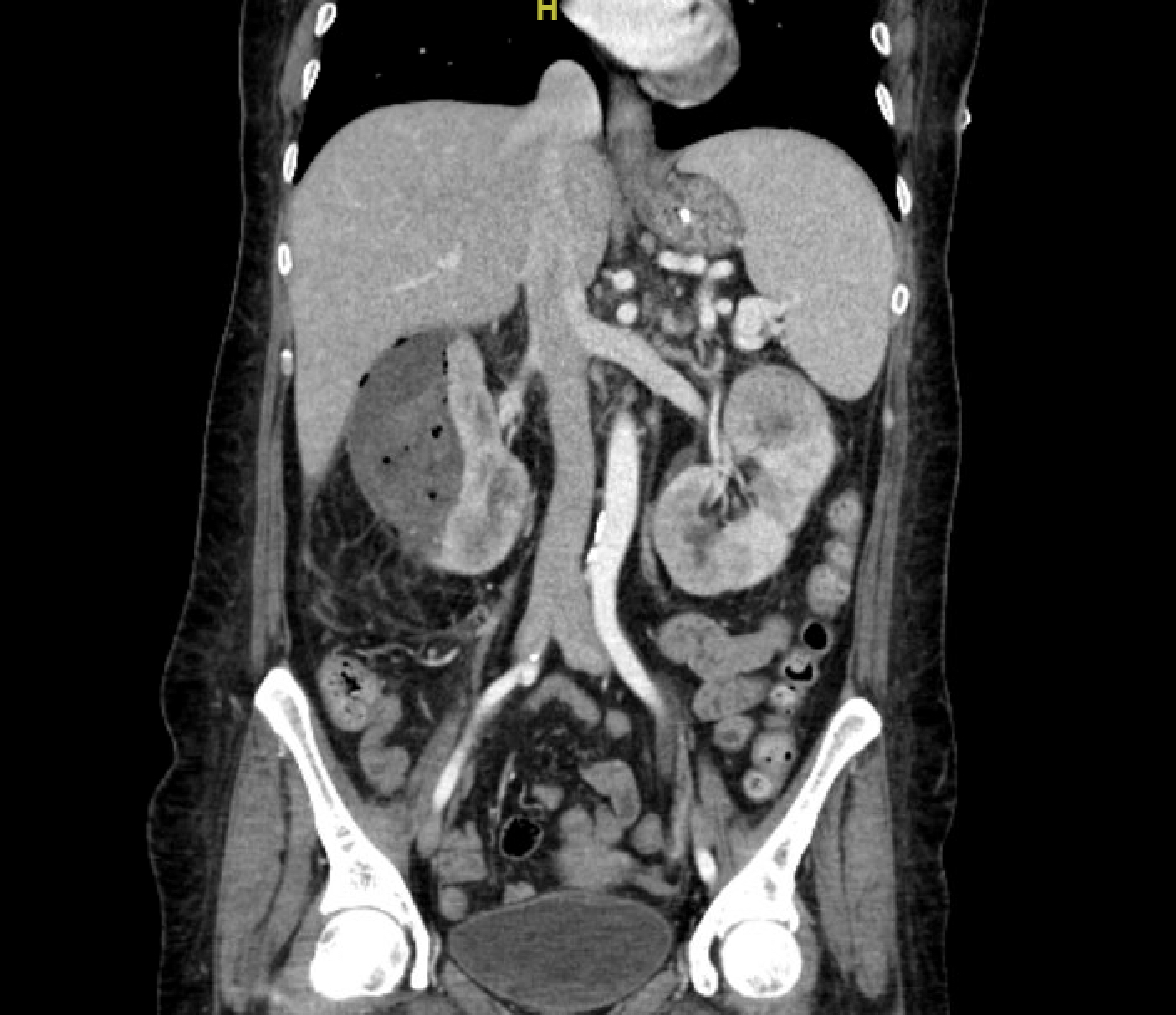

Supplement: Supplementary file 2 [file JETem-8-3-V14-supp2.jpg]

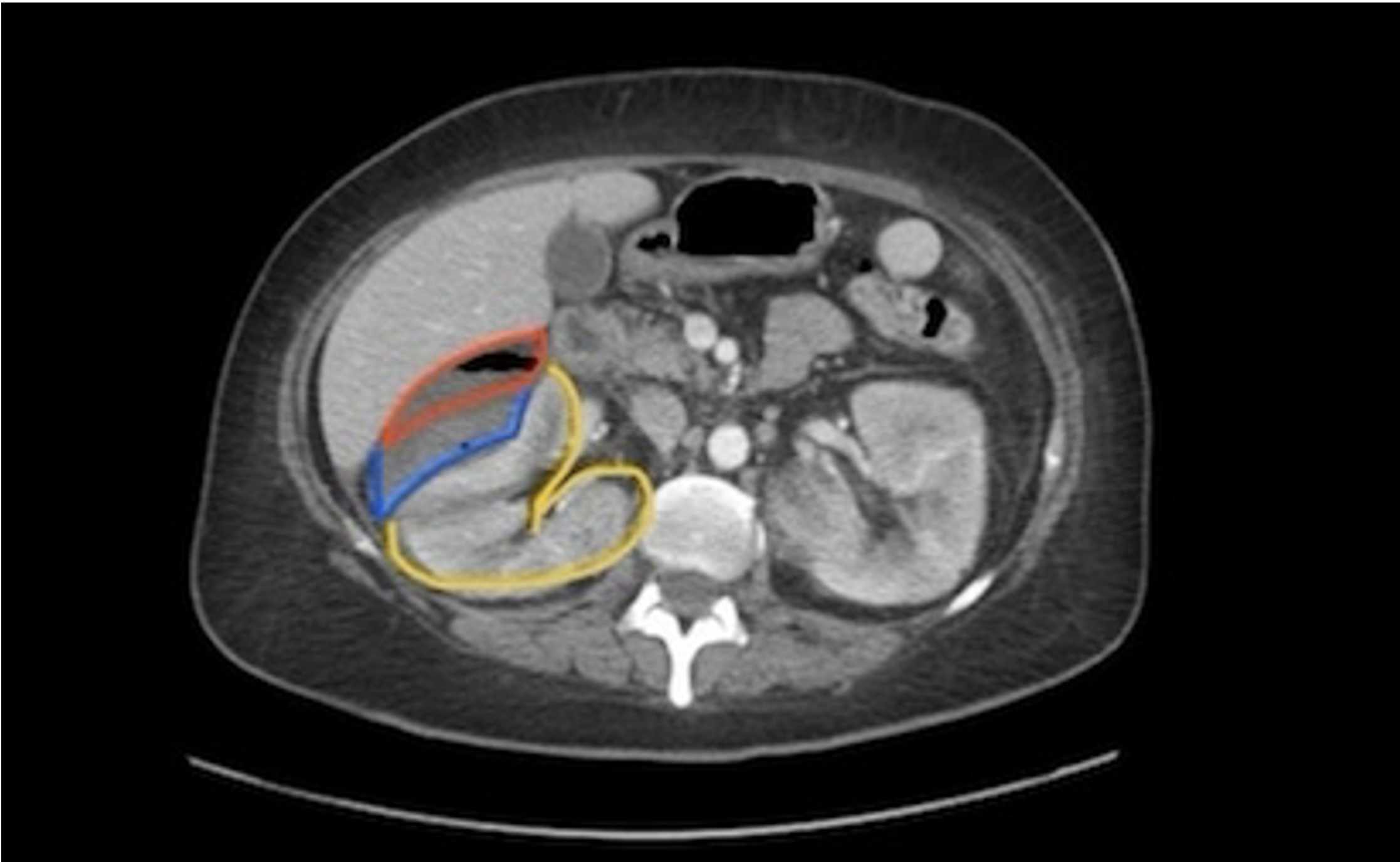

Supplement: Supplementary file 3 [file JETem-8-3-V14-supp3.jpg]

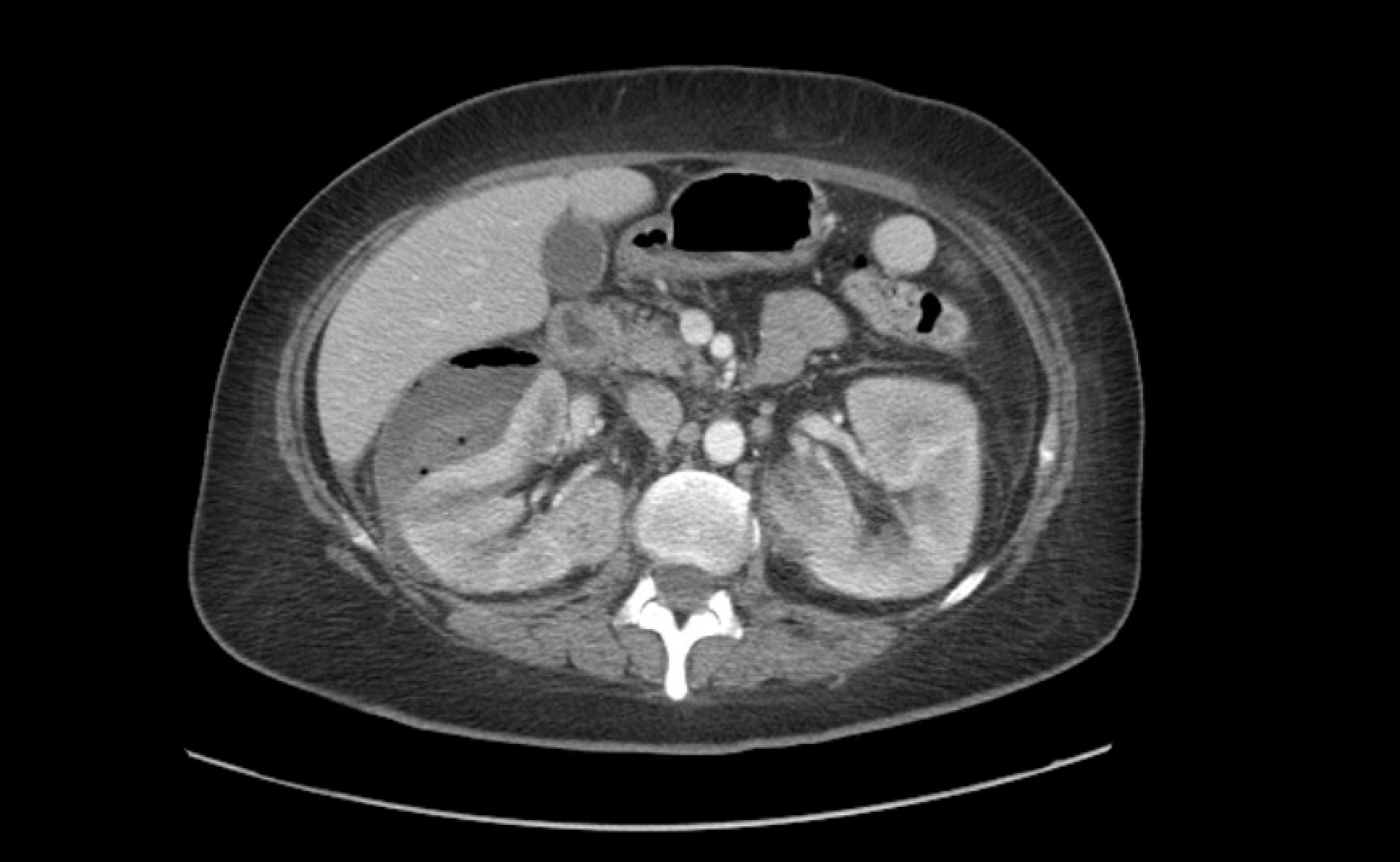

Supplement: Supplementary file 4 [file JETem-8-3-V14-supp4.jpg]
